# Multiple myeloma presented as acute renal failure

**Published:** 2013-07-01

**Authors:** Ali Momeni, Ali Hajigholami, Hamid Nasri

**Affiliations:** ^1^Division of Nephrology, Department of Internal Medicine, Shahrekord University of Medical Sciences, Shahrekord, Iran; ^2^Division of Hematology, Department of Internal, Medicine, Shahrekord, University of Medical Sciences, Shahrekord, Iran; ^3^Department of Nephrology, Division of Nephropathology, Isfahan University of Medical Sciences, Isfahan, Iran

**Keywords:** Multiple myeloma, Kidney, Renal failure

**Implication for health policy/practice/research/medical education:**

It seems that multiple myeloma in not very rare in young population. Multiple myeloma (MM) in this age range may be with unusual presentation or atypical course, hence we suggest to evaluate and rule-out of MM in acute renal failure patients with unknown cause at any age.

Multiple myeloma (MM) is a plasma cell dyscrasia and it most often occurs in old age adults. The mean age of the patients at diagnosis is 66 years old and only few percent of patients are younger than 40 years old (1,2). At the time of diagnosis, about 3% of MM patients have no M-protein in their serum or urine, based on immunofixation method (1-3). Approximately 50% of MM patients have at least some degrees of renal insufficiency and about 9% of them require dialysis because of severe renal failure (1-3). During the course of disease, approximately 20% of the patients will develop progressive renal failure. There are several causes for renal failure in MM such as hypercalcemia, hyperuricemia, Bence Jones cast nephropathy, light chain deposition disease, amyloidosis and interstitial nephritis. Bence Jones cast nephropathy is the most common cause of significant renal dysfunction. Light chain deposition disease and amyloidosis should be suspected if nephrotic range proteinuria is present. The urine dipstick for protein is positive in amyloidosis and negative in myeloma cast nephropathy (2-5). Severity of renal disease has correlation with patient’s survival. In the study of Winearls, one-year patient survival, at disease presentation were 80% and 50% in patients with a plasma creatinine below than 1.5 mg/dl versus above 2.3 mg/dl, respectively (6).

Recently, we had some MM patients with age under 40 and various unusual presentations that is described briefly as below: The first patient was a 39-year-old man who presented with acute renal failure, normal urine electrophoresis and normal skull X-ray. In renal biopsy of the patient, acute tubulointerstitial nephritis was reported (Figure 1). Also in bone marrow aspiration near, 90% plasma cells was seen (7). The second case was a 35-year-old female who was admitted in emergency ward for evaluation of acute renal failure. In renal biopsy Bence Jones cast nephropathy was diagnosed (Figure 2). Treatment of multiple myeloma was started and renal function was improved as she has normal serum creatinine and is in the waiting list of bone marrow transplantation. The third case was a 39-year-old man, who presented with hypercalcemia and acute renal failure. After the beginning of MM treatment, serum creatinine decreased and the patients was nominated for bone marrow transplantation, however after a few months, flare-up of disease was occurred due to pelvic mass consistent with plasmacytoma and now he is undergoing chemotherapy treatment (Figure 3). As noted above, it seems that MM in not very rare in young population (1-6). MM in this age range may be with unusual presentation or atypical course, hence we suggest to evaluate and rule-out of MM in acute renal failure patients with unknown cause at any age.

**Figure 1:**
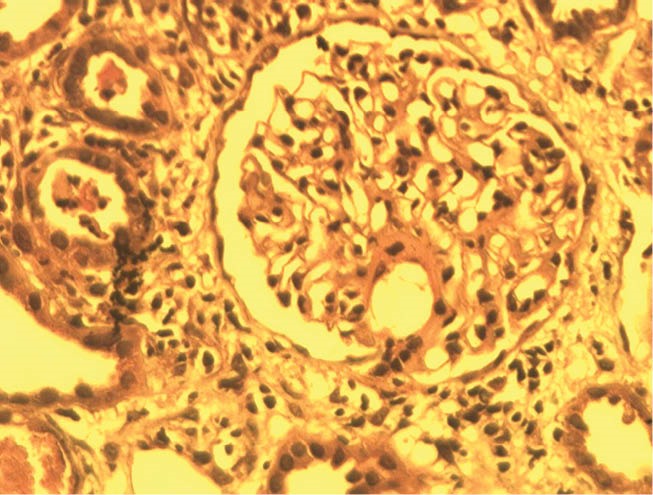
Acute tubulointerstitial nephritis in case 1

**Figure 2:**
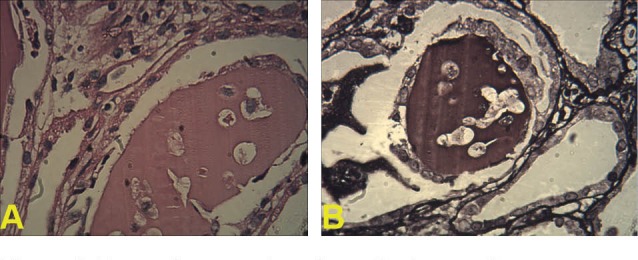
Bence Jones cast nephropathy in case 2

**Figure 3:**
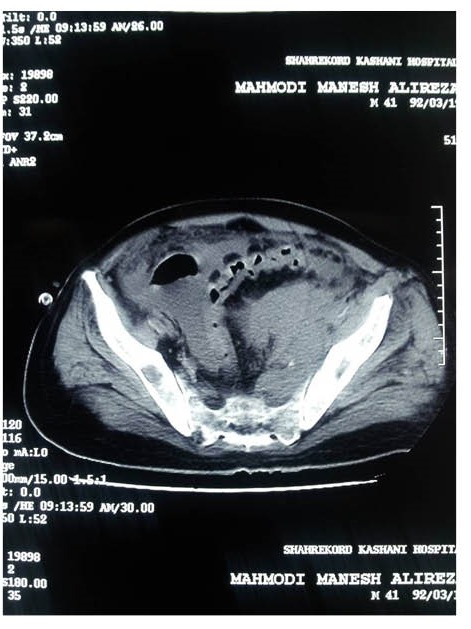
Pelvic mass in case 3

## Authors’ contributions

AM and AH wrote the paper. HN reported the pathology of the cases and edited the paper.

## Conflict of interests

The authors declared no competing interests.

## Ethical considerations

Ethical issues (including plagiarism, misconduct, data fabrication, falsification, double publication or submission, redundancy) have been completely observed by the authors.

## Funding/Support

None.
